# Linking GPS and travel diary data using sequence alignment in a study of children's independent mobility

**DOI:** 10.1186/1476-072X-10-64

**Published:** 2011-12-05

**Authors:** Suzanne Mavoa, Melody Oliver, Karen Witten, Hannah M Badland

**Affiliations:** 1 SHORE and Whariki Research Centre, School of Public Health, Massey University, Auckland, New Zealand; 2 National Institute for Public Health and Mental Health Research, Auckland University of Technology, Auckland, New Zealand; 3 Centre for Physical Activity and Nutrition, Auckland University of Technology, Auckland, New Zealand; 4 The McCaughey Centre: VicHealth Centre for the Promotion of Mental Health and Community Wellbeing, School of Population Health, the University of Melbourne, Melbourne, Australia

**Keywords:** GPS, travel diaries, sequence alignment

## Abstract

**Background:**

Global positioning systems (**GPS**) are increasingly being used in health research to determine the location of study participants. Combining GPS data with data collected via travel/activity diaries allows researchers to assess where people travel in conjunction with data about trip purpose and accompaniment. However, linking GPS and diary data is problematic and to date the only method has been to match the two datasets manually, which is time consuming and unlikely to be practical for larger data sets. This paper assesses the feasibility of a new sequence alignment method of linking GPS and travel diary data in comparison with the manual matching method.

**Methods:**

GPS and travel diary data obtained from a study of children's independent mobility were linked using sequence alignment algorithms to test the proof of concept. Travel diaries were assessed for quality by counting the number of errors and inconsistencies in each participant's set of diaries. The success of the sequence alignment method was compared for higher versus lower quality travel diaries, and for accompanied versus unaccompanied trips. Time taken and percentage of trips matched were compared for the sequence alignment method and the manual method.

**Results:**

The sequence alignment method matched 61.9% of all trips. Higher quality travel diaries were associated with higher match rates in both the sequence alignment and manual matching methods. The sequence alignment method performed almost as well as the manual method and was an order of magnitude faster. However, the sequence alignment method was less successful at fully matching trips and at matching unaccompanied trips.

**Conclusions:**

Sequence alignment is a promising method of linking GPS and travel diary data in large population datasets, especially if limitations in the trip detection algorithm are addressed.

## Background

Global positioning systems (**GPS**) are increasingly used in health research to determine the location of study participants. Despite the utility of GPS in providing objective information about where participants travel and at what time, it does not reveal the purpose or meaning of their movement; what they were doing, who they were with, or why they were in that location. Therefore, in most health related studies GPS data is combined with other datasets that provide additional information about the participants, their behaviour and their immediate environment. For example, GPS data is commonly augmented with Geographic Information Systems (**GIS**) data to identify and characterise locations visited. GPS data can also be linked with data collected using mobile monitors such as accelerometers and air pollution monitors to provide spatio-temporal information about participants and the environment. Data from these objective measures are easily linked using spatial coordinates and timestamps from internal clocks (e.g., [1]).

GPS data can also be combined with information obtained via self-report which is useful in collecting information such as participant perceptions, trip purpose, and accompaniment (i.e. who is with the participant). However, a problem with combining GPS and self-report data is the difficulty in linking the two datasets. Diaries (travel or activity) are perhaps the most straightforward self-report dataset to link with GPS data because they are a timed sequential list of trips or activities undertaken, which means that it is theoretically possible to link the datasets using timestamps. However, being self-reported, diary data is subject to recall biases and times are unlikely to precisely match the GPS times due to participants' not recalling accurate times, and differences in the internal GPS clock and the watches/clocks used by participants. The likely time mismatches mean that it is difficult to use timestamps to automatically link GPS data with diary data, which is a problem for researchers wanting to combine these two datasets.

To our knowledge, linking GPS and diary data through manual matching is the only method that has been used to date [2-4]. Of these studies, the CAPABLE (Children's Activities, Perceptions and Behaviour in the Local Environment) project, a study of children's independent mobility (**IM**) [2], is the only study so far to report on how GPS and travel diary data were linked. However, even here details on the methodology used are scant. The authors reported difficulties in reconciling GPS and activity data sets with the children's diaries, and consistent with reports by other researchers [3,5], they found under-reporting of trips in travel diaries [2]. In addition, 'considerable effort' was required to equate trip times reported in trip diaries with those indicated in GPS records.

As far as we can deduce, manual matching works well, but is vulnerable to operator error and subjectivity. Manual matching is also labour intensive, which can make it prohibitively expensive for large studies.

To address the problem of linking GPS and diary data in a large study population and to avoid the issue of inaccurately entered travel diary times we developed a partially automated method of linking the datasets using sequence alignment algorithms. Sequence alignment is based on the principle of comparing sequences of strings [6]. It was developed in the 1980s for use in the natural sciences to analyse deoxyribonucleic acid (**DNA**) sequences. Since then its use has expanded to other fields including transportation and urban planning [7,8], sociology [9,10], the analysis of sketch maps [11], and tourist behaviour [12]. Sequence alignment methods are computational and, through automation of the process, have the potential to reduce the time taken to link GPS and travel diary. In addition, automating the linking of GPS and diary data (i.e., writing code) provides the added benefit of documenting the process and making it objective, repeatable, and replicable.

This paper assesses the feasibility of a new method of linking GPS and travel diary data in comparison to the only existing alternative method (i.e. manual matching). This is done in the context of a study of children's IM. We begin with a description of the sequence alignment method. Next, the feasibility and utility of this method is assessed by comparing the time taken and trip match rates of the manual and sequence alignment methods of linking GPS and travel diary data. The effect of higher and lower quality travel diary data on the match rate are also assessed. Finally, the results and the practicalities of using sequence alignment to link GPS and travel diary data are discussed.

## Methods

### Dataset

We used a subsample of data from Kids in the City, a study of IM in children aged 9 - 11 years in six neighbourhoods in Auckland, New Zealand [13]. The subsample used in this series of analyses comprises seven day GPS and travel diary data for 40 children from two schools (School A, School B).

### School and participant selection

The schools selected were located in neighbourhoods with differing socio-economic status and walkability characteristics (Table 1). Neighbourhood socio-economic status was determined by the school decile rating, a measure of the socio-economic position of households in a school's catchment area derived from New Zealand Census data. Neighbourhood walkability was determined by calculating a walkability index comprising dwelling density, street connectivity, land use mix and retail floor area ratio [14], and this index was applied to the neighbourhood where the two schools were located. We purposely selected schools with varying quality travel diary data - as reported by the research assistants responsible for data collection - because we were interested in how travel diary quality might affect the success of the sequence alignment method. Twenty children from each school were randomly selected from the total number of participants at each school.

**Table 1:** Characteristics of the dataset.

	School A (n = 20)	School B (n = 20)
School decile*	1	4

Neighbourhood walkability	mid	higher

Travel diary quality	lower	higher

Total number of trips recorded in travel diaries	736	468

Total number of GPS points	2,017,873	1,666,521

* an indicator of socio-economic status

### Data collection

Data collection occurred between March and June 2011. Children wore QStarz BT-Q1000 or BT-Q1000XT GPS units (Qstarz International Inc., Taiwan) on a belt for seven consecutive days. The units were configured to log data every 10 s. Children completed travel diaries for the same period. These were collected and checked with the children every school day during data collection. Further details on data collection are available in Oliver et al. [13].

### Travel diary quality assessment

The quality of the travel diaries was assessed by counting the number of errors and inconsistencies evident in each child's set of diaries. Indicators of low quality were: missing data, data that had been obviously adjusted or entered by a researcher, and inconsistencies such as a child travelling to a park but not recording a trip back home. The number of indicators of low quality were summed, resulting in a diary quality score for each child, with higher scores representing lower quality diaries.

The travel diary quality scores ranged from 0 - 25. The 17 children with travel diary quality scores greater than 4 were assigned a quality cateqory of 'lower', while the 23 children with quality scores less than or equal to 4 were assigned a quality category of 'higher'.

### Overview of the sequence alignment method

**Figure 1:**
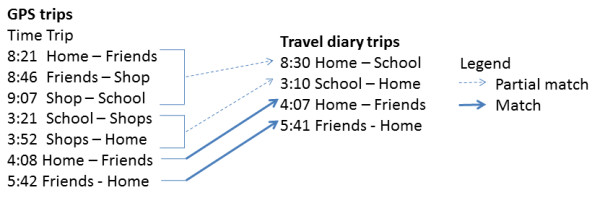
**The concept behind the sequence alignment method**.

The sequence alignment method described below matched GPS and travel diary datasets according to the sequence of trips. Trips were matched based on their origin and destination locations and on the order of trips in a day as established by the travel diary. For example, in Figure 1 there are two 'Home - Friends' trips identified in the GPS dataset for a nominated child, yet only one in the travel diary dataset. Using the sequence alignment method the second GPS 'Home - Friends' trip would be matched with the travel diary 'Home - Friends' trip because it occurred after the 'Home - School' trips. By relying on the relative order of the trips it is not necessary to use the times entered in the travel diaries.

The method matches trip chains with two links. For example, the GPS trips from 'School - Shops' and 'Shops - Home' are matched with the travel diary trip 'School - Home'. The method also distinguishes between full and partial matches. A full match is where both the origin and destination locations match. A partial match is where only one of the origin or destination locations match.

### Implementation

GIS software, ArcGIS v.9.3 (ESRI, Redlands), and open source statistical computing software, *R *(R Foundation for Statistical Computing, Vienna, Austria), were used to implement the sequence alignment method. Custom scripts were written in the Python [15] and *R *scripting languages. These scripts are available from the lead author upon request.

**Figure 2:**
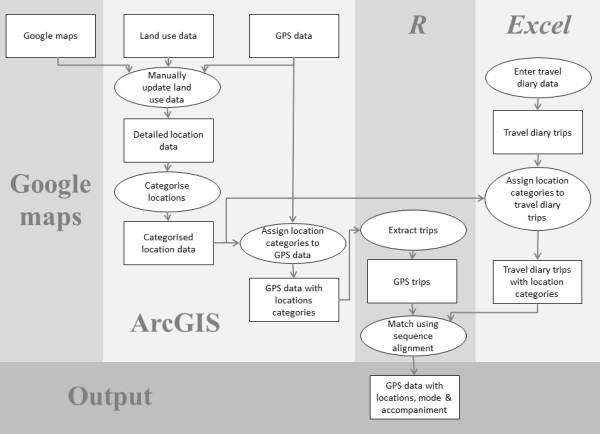
**Sequence alignment implementation process**.

The implementation process is illustrated in Figure 2. In the absence of established protocols to clean GPS data, we cleaned the data by removing data points with speeds greater than 160 km/h, horizontal dilution of precision (**HDOP**) values greater than five, and data points with less than four visible satellites. HDOP is a factor in determining the horizontal accuracy of the GPS data and relates to how the GPS satellites are positioned in the sky [16].

GPS data were imported into ArcGIS along with land use data supplied by two territorial local authorities. The supplied land use datasets comprised six common locations: roads, residential, commercial, industrial, parks, and schools. We improved the land use datasets by visually inspecting all GPS tracks, checking locations coincident with these tracks against Google Maps and Google Streetview (Google Inc., Mountain View, CA), and manually updating the land use dataset where more detailed location data were available. As a result, the improved land use dataset was based on all locations where the 40 children in the sample recorded spending time. The locations in the improved land use dataset are listed in Table 2. Separate GIS datasets representing the home and school locations for each child were created by extracting the home and school land parcel polygons from a land parcel database.

**Table 2:** Locations, location categories and location codes for the example dataset.

Location category	Location	Location code
Home	Home	A

School (the school attended by the children)	School	B

Residential (excluding the home address, this will cover trips to visit friends and family)	Residential Residential driveways Retirement home	C

Shops	Petrol stations Shops Shopping centres	D

Open space	Beaches Public open space (parks) Unofficial open space (e.g. empty lots)	E

Sports facilities	Indoor sports facilities Outdoor sports facilities Swimming pools	F

Church	Church	G

Medical (including doctors, medical clinics and hospitals)	Medical	H

Other schools (schools not attended by the children)	Schools	I

Other (uncommon and unknown locations)	Accommodation (e.g. hotels and motels) Airport Car parks Industrial Other Preschools Tertiary institute Water	K

The locations in the improved land use dataset were assigned location categories based on the available data, and on the specificity of destination information provided by the children in their travel diaries (Table 2). To ensure the data were comparable between children we used the coarsest level of detail reported in the travel diaries. For example, we classified all types of shopping locations into a single 'Shops' category as opposed to several categories (e.g., convenience store, supermarket, shopping centre). We aggregated uncommon locations, such as the airport, into a single 'Other' category in order to minimise the number of unique location categories. This was done to keep the first trial of the sequence alignment method as simple as possible.

Next, each GPS data point was assigned a location category and an associated location code based on the improved land use dataset (Table 2). We used letters of the alphabet as location codes in order to allow easier implementation of the sequence alignment algorithm.

The GPS data were exported to *R*, where custom scripts were used to extract trips. For the purposes of testing the sequence alignment method a basic trip detection process was employed. A trip was defined as the period of travel along street networks between two stops at different locations. We defined a stop using a dwell time of 120 seconds, which is the most commonly used dwell time in trip detection literature [17].

The trip detection process resulted in a list of GPS trip sequences for each child on each monitored day. Trips were assigned codes based on the origin and destination location codes. For example, a 'Home - School' trip was converted to 'Ab' and a 'School - Shops' trip was converted to 'Bd.'

Travel diaries were entered in an Excel spreadsheet as trip sequences and trip codes. Origins and destinations were standardised to match the location categories (Table 2). Trip sequences from the travel diary spreadsheets were imported into *R*.

Finally, the sequence of GPS trips and the sequence of travel diary trips were matched using a custom script. This script implemented the Needleman-Wunsch algorithm which performs a global sequence alignment on two sequences [18]. The script also allowed for full and partial trip matching in order to accommodate the non-reporting of chained trips in travel diaries. A full match was identified where both the origin and destination location categories matched, whereas a partial match was where only one of either origin or destination location categories matched. In the version of the script reported here, partial matches were only made where two trips equated to one trip. For example in Figure 1, only the 'Home - Friends' GPS trip at 8:21 would be partially matched with the 'Home - School' travel diary trip at 8:30, whereas *both *the 'School - Shop's GPS trip at 3:21 and the 'Shops - Home' trip at 3:52 would be partially matched with the 'School - Home' travel diary trip at 3:10.

**Figure 3:**
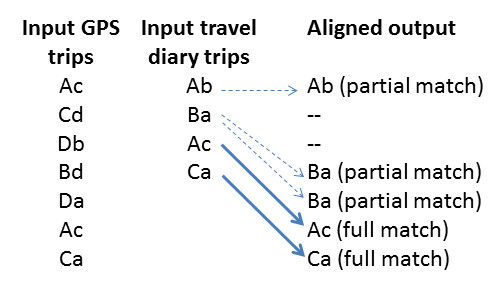
**Example input and output from the sequence alignment process**.

A GPS trip sequence and a travel diary trip sequence in the form of character codes were used as input into the script. Where the trips were fully or partially matched, data about travel mode and accompaniment were automatically copied from the travel diary dataset to the GPS dataset (Figure 3).

### Matching the GPS and travel diary trip sequences manually

In order to provide a comparison between the sequence alignment method and the manual method, we manually matched a subset of the example dataset. Five participants were randomly selected from each school. The GPS and travel diary data across all days for these ten participants were manually matched by a researcher. GPS data were viewed in ArcGIS and visually compared with information from the travel diary. GPS trips were identified and data about travel mode and accompaniment were manually assigned to GPS points where the GPS and travel diary trips matched (i.e., same date, similar time, and similar location categories). Full and partial matches were identified. In cases where there were no travel diary data available, GPS trips were derived but were not assigned trip information.

### Comparison measures

Two comparison measures were collected during the matching process in order to compare the sequence alignment method and the manual method. The first was *number of trips matched*. This was a count of the number of full, partial, and unmatched trips for each participant using the sequence alignment and manual matching methods.

The second comparison measure was *time taken to match*. For the sequence alignment method, time was recorded for the following processes: preparing GPS data, converting the trips into trip sequences, converting travel diary data into trip sequences, matching the GPS and travel diary sequences, and copying information from the linked travel diary to the GPS data points. This time was separated into 'manual' - where a researcher was required to complete the entire task, or 'automated' - where a researcher was only required to start a script and was then able to leave the processing unattended. All automated scripts ran on a computer with an Intel Core i5 CPU processor (3.73 GHz), 4 GB RAM and a 32-bit Windows Operating System. For the manual method the time taken to manually match GPS and travel diary data for each participant on each day of the week was recorded.

### Statistical analyses

*R *was used to perform chi-squared tests of independence to determine differences between schools, and between the two matching methods.

## Results

### Sequence alignment method

Matching GPS and travel diary data for 40 children using the sequence alignment method resulted in 61.9% of the travel diary trips being fully (29.5%) or partially (32.4%) matched (Table 3). The overall match rate for higher quality travel diaries (68.2%) was greater than for lower quality travel diaries (55.9%). Higher quality travel diaries had a greater percentage of full matches than lower quality travel diaries (χ^2 ^= 6.518, df = 2, *p *= 0.038).

**Table 3:** Results from the sequence alignment matching method.

	Travel Diary Quality		Total
	Lower	Higher	
Number of children	17	23	40

**Number of trips**			

Total travel diary trips	553	617	1170

Total GPS trips	558	827	1385

**Number of matched trips**			

Fully matched trips (% of all travel diary trips)	126 (22.8%)	219 (35.5%)	345 (29.5%)

Partially matched trips (% of all travel diary trips)	183 (33.1%)	202 (32.7%)	379 (32.4%)

**Number of unmatched trips**			

Unmatched travel diary trips (% of all travel diary trips)	244 (44.1%)	196 (31.8%)	446 (38.1%)

### Comparing the sequence alignment and manual methods

Comparing the results from the sequence alignment and manual methods for 10 participants, the sequence alignment method resulted in 32.1% fully matched trips and 30.1% partially matched trips, whereas the manual method resulted in 48.2% fully matched trips and 22.9% partially matched trips (Table 4). The manual method was significantly more successful at fully matching trips, and in matching a greater number of trips than the sequence alignment method (χ^2 ^= 16.747, df = 2, *p *< 0.001).

**Table 4:** Number of trips matched using the sequence alignment and manual methods.

	Travel Diary Quality				Total	
	Lower		Higher			
	
	Sequence alignment	Manual	Sequence alignment	Manual	Sequence alignment	Manual
Number of children	6	6	4	4	10	10

**Number of trips**						

Total travel diary trips	220	208	92	97	312	305

Total GPS trips	189	199	125	93	314	292

**Number of matched trips**						

Fully matched trips (% of all travel diary trips)	45 (20.5%)	80 (38.5%)	55 (59.8%)	67 (69.1%)	100 (32.1%)	147 (48.2%)

Partially matched trips (% of all travel diary trips)	67 (30.5%)	53 (25.5%)	27 (29.3%)	17 (17.5%)	94 (30.1%)	70 (22.9%)

**Number of unmatched trips**						

Unmatched travel diary trips (% of all travel diary trips)	108 (49.0%)	75 (36.0%)	10 (10.9%)	13 (13.4%)	118 (37.8%)	88 (28.9%)

We investigated whether trips listed as independent in the travel diaries were equally matched using both methods. In the 10 child dataset, 28.9% of trips were independent. Using the sequence alignment method 48.9% of these independent trips were matched, whereas using the manual method that figure rose to 69.3%.

**Figure 4:**
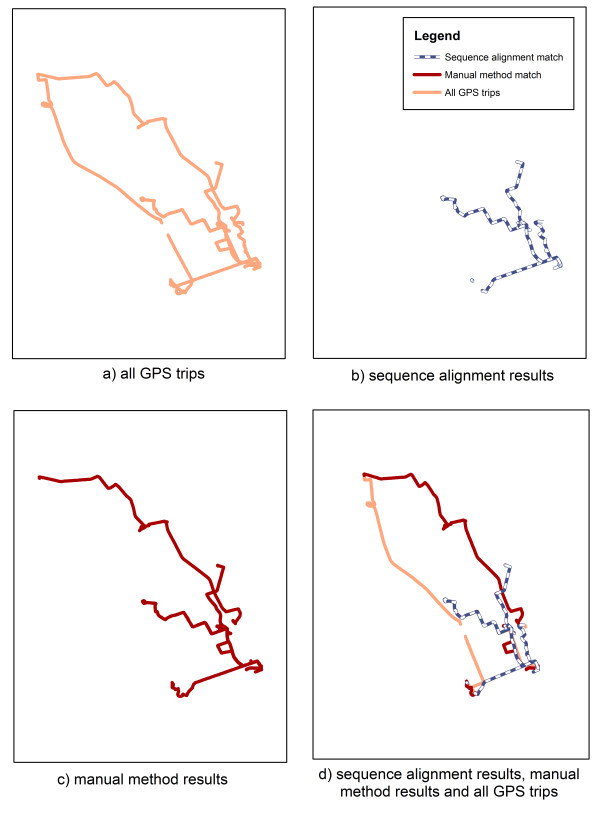
**Results of the two matching methods for one participant over a seven day period**.

We were interested in whether the different methods matched the same trips in the same manner. Figure 4 show the GPS data for a single participant over a seven day period with the results of matching using the two methods. It illustrates that the two different methods (Figure 4b, c) resulted in several identically matched trips, however one trip was matched differently. Based on the results observed in the current investigation, a similar pattern of some identical matches and a few different matches could be expected with the rest of the dataset. This figure also shows that neither method successfully matched all the GPS data (Figure 4a).

Large differences in the time taken to classify trips were found between the two methods. It took approximately 80 minutes to match seven days of GPS and travel diary data for 10 children using the sequence alignment method. However, only 60 minutes of this involved manual work on the part of the researcher as the remaining 20 minutes were automated. The trip detection script took approximately 2 minutes per child, or approximately 20 s per day of GPS data. The sequence alignment script, which performed the sequence alignment and assigned match information to the datasets, took approximately 5 s for 7 days of trips for 10 children. By contrast, it took approximately 1,881 minutes to match the same data using the manual method, with none of this time being automated. We did not include the time taken to prepare the GIS databases of origins and destinations in the time comparison because this database was an essential component in both the sequence alignment and manual methods. Manual preparation of the detailed GIS database of origins and destinations was a time consuming process that took between 30 - 120 minutes per child. However, the first few children took the most time and the process got progressively faster as children from the same school tended to visit the same locations.

## Discussion and conclusion

This paper assessed the feasibility of a new method of linking GPS and travel diary data in comparison to the only existing alternative method (i.e. manual matching). The sequence alignment method successfully matched 61.9% of travel diary trips. This result is encouraging, as the overall match rate for the manual method was only 8.9 percentage points higher. As expected, in both methods lower quality travel diaries were associated with lower match rates, although the manual method was slightly better at matching lower quality diaries. Despite the small difference in the overall match rate, the sequence alignment method did not perform as well as the manual method in fully matching trips. On the other hand, the sequence alignment method was an order of magnitude faster. Given the time savings it is worth considering how the sequence alignment method could be improved to increase the percentage of trips matched.

The main limitation of the sequence alignment method is the trip detection algorithm. Here, there are three main areas for improvement. The first is improving the way trip chains are handled. The sequence alignment method only accommodates two links in a trip chain. For example, two sequential GPS trips of 'Home - Shops' and then 'Shops - School' would be partially matched to a single travel diary trip of 'Home - School.' However, any trips with more than two links would not be matched, resulting in a greater proportion of unmatched trips. Trip chains with more than two links were not included in this version of the sequence alignment method because this was a preliminary investigation of the method's utility. The second area for improvement in the trip detection algorithm concerns off-road trips such as trips through parks and along pedestrian paths. Currently the trip detection algorithm excludes trips that are taken off-road and this would also have contributed to the lower match rate because any off-road trips would not be matched. The third area for improvement relates to the dwell time used to identify trip end points. The trip detection algorithm used 120 s as that is the most common dwell time used in the literature, however this time may not be appropriate for all population groups.

The purpose of the study and the population group being assessed are important considerations when determining the utility of the sequence alignment method. The example data used here came from a study of children's IM where the purpose was to identify GPS trips undertaken independently. Thus, although the results of the sequence alignment method are promising, for this example dataset and purpose, the lower percentage of matches for independent trips are a concern. Addressing the limitation of the trip detection algorithm would improve the percentage of matches for independent trips because the excluded off-road journeys are more likely to be independent than journeys along the street network, which include dependent trips in cars. However, this illustrates that the sequence alignment method may be more appropriate for certain types of trips than others.

The example data also illustrated the importance of the population group in assessing the results from the sequence alignment method. We expect that different population groups will have different mobility behaviours and as a result the sequence alignment method may work better with certain populations. The example data came from a specific population group: children aged 9-10 years. In performing the manual matching we observed that travel diaries did not always adequately capture children's behaviour. This failure may have been the result of the children's conceptions of what constitutes a trip. For example, a child playing around their home might travel between their home, neighbouring homes, and on the footpaths and roads outside their home many times within a short period. This type of behaviour would appear on a GPS trip sequence as: 'Home - Residential', 'Residential - Residential', 'Residential - Home', 'Home - Home', 'Home - Home', whereas in the corresponding travel diary the child is likely either to enter 'Playing outside' or to enter nothing at all if they consider playing in the vicinity of their home to be the same as playing at home. Because the sequence alignment method is based on the conceptualization of travel behaviour as trips, children's play outside the home and around the neighbourhood that is not recorded as a trip cannot be detected by the sequence alignment method. This means that population groups who have a higher proportion of structured travel are more likely to have higher matches in both the sequence alignment and manual methods.

Although the inability of travel diaries to represent unstructured mobility is not a limitation of the sequence alignment method itself, it is an important issue for researchers using travel diaries to assess mobility. Addressing this issue may require rethinking the way in which mobility data is captured. One possibility is to use reduce the emphasis on trips in the diaries, for example using activity diaries that focus the participants on activities undertaken instead of travel diaries that are focused on trips. Another possibility is using a different data collection method. New technologies that enable researchers to collect objective data about aspects of a participant's life, such as who is accompanying them, may eventually make diaries redundant. Visual life-logging technologies, where every day activities are captured by digital cameras, are likely to be a major development in this field. Kelly et al. [19] have identified life-logging as a potential alternative to self-report and direct observation of travel. By capturing images of the environment surrounding a participant it may be possible to determine trip mode, purpose and accompaniment, making life-logging a promising alternative to data collection using diaries. However, this technology is still in the early stages of use in health research and more work is needed before it can be used as a viable alternative to diaries as there are issues concerning ethics, privacy and image processing that need to be addressed.

In conclusion, although the manual method matched a higher percentage of trips, the sequence alignment method was significantly faster. Therefore, we believe that the sequence alignment method is a promising approach for linking travel diary and GPS data, particularly for large datasets where the manual method is likely to be prohibitively time consuming and expensive. Improving the trip detection algorithm is likely to improve the match rate and utility of the sequence alignment method. However, the context of the data being matched is an important consideration and this method may be more or less suitable depending on the population being studied and the question being investigated.

## List of abbreviations

GIS: Geographic information systems; GPS: Global Positioning System; h: hour; HDOP: horizontal dilution of precision; IM: independent mobility; km: kilometres; s: seconds.

## Competing interests

The authors declare that they have no competing interests.

## Authors' contributions

SM conceived and implemented the sequence alignment methodology, analysed the data and wrote the first draft of the manuscript. MO and SM manually matched the GPS and travel diary data. All authors provided critical feedback and read and approved the final manuscript.
